# Downregulation of Caveolin‐2 Is a Novel Mechanism Involved in Activation of Inducible Nitric Oxide Synthase (iNOS) in Mouse Peritoneal Macrophages

**DOI:** 10.1155/mi/9244130

**Published:** 2026-05-25

**Authors:** Sungchan Jang, Grzegorz Sowa

**Affiliations:** ^1^ Department of Medical Pharmacology and Physiology, University of Missouri, Columbia, 65212, Missouri, USA, missouri.edu

**Keywords:** caveolin-2, inducible nitric oxide synthase (iNOS), interferon gamma (IFN-γ), lipopolysaccharide (LPS), macrophages, NF-κB, signal transducer and activator of transcription (STAT1)

## Abstract

Here we report that membrane protein caveolin‐2 (Cav‐2) is regulated during activation of primary mouse peritoneal macrophages and RAW264.7 macrophage cell line by a bacterial lipopolysaccharide (LPS) and a cytokine, interferon gamma (IFN‐γ). We also show that downregulation or loss of Cav‐2 increases expression of inducible nitric oxide (NO) synthase (iNOS) and subsequent NO production in activated macrophages. Treatment with LPS and IFN‐γ downregulated Cav‐2 at the protein and mRNA level. Moreover, LPS‐ and IFN‐γ‐induced downregulation of Cav‐2 inversely correlated with iNOS expression levels. Mechanistically, activation of NF‐κB pathway was responsible for downregulation of Cav‐2 by LPS and IFN‐γ since pharmacological inhibition of NF‐κB activation with pyrrolidine dithiocarbamate (PDTC) prevented Cav‐2 downregulation and iNOS activation. To test if LPS‐ and IFN‐γ‐induced downregulation of Cav‐2 could possibly be involved in macrophage activation, we used a combination of siRNA knockdown and genetic deletion/knockout (KO) approaches. Remarkably, reduction of Cav‐2 using siRNA approach resulted in enhanced STAT1 phosphorylation, iNOS expression, and increased NO production in LPS‐ and LPS plus IFN‐γ‐stimulated macrophages. Conversely, genetic deletion of Cav‐2 robustly enhanced IFN‐γ‐stimulated iNOS expression and subsequent NO production. Overall, our data suggest that Cav‐2 is not only regulated during macrophage activation, but it also may be an important physiological regulator of macrophage activation via preventing excessive STAT1 signaling and iNOS stimulation.

## 1. Introduction

Caveolins, in particular the mostly studied caveolin‐1 (Cav‐1) play numerous important functions [1]. It has been well established that in addition to being a key structural protein that organizes caveolar structures, Cav‐1 is involved in regulating various aspects of cell signaling and function [1, 2]. In contrast to the extensively studied Cav‐1, the role of caveolin‐2 (Cav‐2) is far less understood. Nevertheless, recent reports suggest several novel functions for Cav‐2, which are often tissue and cell specific and sometimes opposite to respective functions of Cav‐1 [3]. For example, Cav‐2 has been shown to regulate caveolae formation in certain epithelial cell types [4, 5], endothelial cell proliferation [6, 7], migration of renal cell carcinoma [8], TGF‐β signaling and function in endothelial cells [9, 10], insulin signaling in fibroblasts [11], pulmonary fibrosis in mice [12], bacterial invasion of epithelial cells in vitro [13–17], sepsis in mice induced by a high dose of lipopolysaccharide (LPS) [18], tumor angiogenesis [19], antitumor immune response [20], or in postischemic tissue injury leukocyte‐endothelial adhesive interactions [21].

Nitric oxide (NO) is a gaseous signaling molecule regulating various physiological and pathophysiological processes. The production of NO is increased during inflammation, and it is known to act as a regulatory and proinflammatory modulator in several inflammatory diseases [22, 23]. NO can be synthesized by three NO synthase (NOS) enzymes: endothelial NOS (eNOS), neuronal NOS (nNOS), and inducible NOS (iNOS). However, only expression of iNOS is induced in response to, for example, bacterial products such as the major component of the bacterial cell wall of Gram‐negative bacteria, LPS and proinflammatory cytokines such as interferon gamma (IFN‐γ) and synthesizes high levels of NO for an extended period of time [23, 24]. Macrophage iNOS‐derived NO is particularly important in innate immunity and inflammation [23]. While the high levels of iNOS‐produced NO are beneficial for microbicidal, antiviral, antiparasitic, and antitumoral responses, abnormal iNOS induction may have harmful effects and is involved in the pathophysiology of numerous diseases such as asthma, arthritis, multiple sclerosis, colitis, psoriasis, neurodegenerative diseases, tumor development, transplant rejection, or septic shock [25]. Thus, understanding the mechanisms controlling iNOS induction and activity in macrophages is essential.

Although expression of Cav‐2 has previously been reported in macrophages [26–28] and we have previously shown that global deficiency in Cav‐2 increased numbers of M1‐polarized macrophages in the tumor microenvironment prior to tumor regression [20], little is known about regulation and direct involvement of Cav‐2 in macrophage function, including macrophage activation in response to LPS or cytokines. The goal of this study was to examine if the Cav‐2 expression level is regulated by classical proinflammatory stimuli capable of macrophage activation and if Cav‐2 regulates macrophage activation manifested by iNOS expression and subsequent NO production in primary murine peritoneal macrophages and in a well‐characterized murine macrophage cell line RAW264.7. Our data show that Cav‐2 is downregulated by LPS and IFN‐γ via the NF‐κB‐dependent pathway and that reduction of Cav‐2 with the RNAi approach as well as genetic deletion of Cav‐2 enhances iNOS expression and NO production in vitro.

## 2. Materials and Methods

### 2.1. Antibodies

Antibodies against total Cav‐2, Cav‐1, and phosphotyrosine 701 STAT1 (P‐STAT1) were from BD Transduction Labs; Antibody to phosphoserine 536 NF‐κB p65 (P‐NF‐κB p65) was from Cell Signaling. Antibodies to iNOS and glyceraldehyde‐3‐phosphate dehydrogenase (GAPDH) were obtained from Santa Cruz Biotech.

### 2.2. Cells

Mouse resident peritoneal macrophages were isolated from 3 to 4‐month‐old wild type (WT) and Cav‐2 KO C57BL/6 mice as previously described [29] with minor modifications. Briefly, mice euthanized by CO_2_ inhalation were i.p. injected with 10 mL of sterile DPBS. Pooled peritoneal cells collected from four to six mice were washed, resuspended in culture medium, and seeded onto 24‐well cell culture treated plates at 5 x 10^5^ cells/well. Adherent cells (macrophages) were purified from the remaining peritoneal cells isolated by incubating the cells for 2 h at 37°C, 5% CO_2_, followed by vigorous shaking of the plate and washing the wells three times to remove nonadherent cells. Cultures were maintained at 37°C and 5% CO_2_ in a humidified Sanyo incubator. Macrophages were cultured in 1:1 mixture of DMEM (Invitrogen) and F‐12 Ham’s Nutrient Mixture (HyClone) plus 10% FBS (HyClone), 1% Pen/Strep, 100 mM sodium pyruvate, 1% (v/v) MEM NEAA, and 200 mML‐glutamine (all from Invitrogen). Mouse macrophage cell line RAW264.7 was obtained from ATCC and cultured in DMEM high glucose (Invitrogen) supplemented with 10% HI FBS (Hyclone) and 1% (v/v) pen/strep. Where indicated cells were stimulated with LPS from *Escherichia coli* 0128:B12 (Sigma), recombinant murine IFN‐γ (Peprotech), and a combination of LPS and IFN‐γ. To determine involvement of the NF‐κB pathway in regulation of Cav‐2 by LPS and IFN‐γ, RAW264.7 were pretreated for 1 h with pyrrolidine dithiocarbamate (PDTC) at 10, 20, and 50 μM followed by additional treatment in the absence or presence of LPS and IFN‐γ for up to 20 h.

### 2.3. SDS‐PAGE and Immunoblotting

SDS‐PAGE and immunoblotting were performed as described previously [10]. Briefly, cells were lysed in Laemmli SDS loading buffer, followed by boiling for 5 min. An equal protein amount was loaded on SDS‐PAGE, and proteins were electrotransferred onto nitrocellulose membranes. The membranes were washed in tris‐buffered saline with 0.1% Tween, blocked in 5% milk, and incubated with the appropriate primary antibodies diluted 1:1000–1:20,000 at 4°C overnight, followed by incubation with horseradish peroxidase labeled secondary antibodies diluted 1:10,000, and developed by enhanced chemiluminescence. The densitometric values for the indicated proteins were determined using Image J (NIH). The data are expressed as densitometric ratios of the expression levels of specific proteins to GAPDH assessed from one representative out of three total experiments.

### 2.4. RNA Isolation and Quantification of Specific Gene Expression by Quantitative Real Time PCR

Total RNA was isolated from RAW264.7 using the TRI reagent (Sigma). 1 μg of isolated RNA was then reverse transcribed into cDNA using the SuperScript First‐Strand Synthesis System for RT‐PCR (Invitrogen). Relative expression levels of Cav‐2 and iNOS were determined by quantitative PCR. GAPDH was used as the housekeeping gene, and gene expression was measured using a QuantiTect SYBR Green RT‐PCR kit (Qiagen) with the BIO‐RAD iQ5 Optical Module plus a BIO‐RAD iCycler Thermal Cycler. The following primers were used for the amplification of mouse: Cav‐2 (RefSeq ID: NM_016900): forward, 5′‐ CTTCATTGCGGGTATCCTGT‐3′ and reverse, 5′‐ATATTGTCTGCACGGAAGGC‐3′; iNOS (RefSeq ID: NM_ 010927): forward, 5′‐ GTCGATGTCACATGCAGCTT‐3′ and reverse, 5′‐ GAAGAAAACCCCTTGTGCTG‐3′; GAPDH (RefSeq ID: NM_008084): forward, 5′‐CGTCCCGTAGACAAAATGGT‐3′ and reverse, 5′‐TTGATGGCAACAATCTCCAC‐3′. Thermal conditions were 15 min at 95°C and 40 cycles of 10 s at 95°C, 30 s at 52°C, and 30 s at 72°C. Values are calculated based on the amount of target mRNA normalized to the endogenous reference GAPDH mRNA based on the following equation: 2^−ΔΔCt^. Data are expressed as fold change by LPS and IFN‐γ relative to control samples at each time point and represented as mean ± S.D. of three replications from one representative out of three total experiments.

### 2.5. Nitrite Accumulation

The ability of the cells to produce NO was determined by colorimetric (570 nm) measuring the accumulation of nitrite in culture media, a stable metabolite of NO using Griess Reagent System (Promega; Cat.# G2930) according to manufacturer’s instructions. The data are expressed as the mean nitrite concentration ± S.D. (*n* = 3) from one representative out of a total of three experiments.

### 2.6. siRNA Knockdown

RAW264.7 were transfected with 20 nM of control (nonsilencing) and mouse Cav‐2 specific stealth siRNA (Invitrogen corp.) using RNAiMAX transfection reagent (Invitrogen corp.) according to manufacturer’s protocol. Three days after starting transfection, cells were stimulated with LPS and IFN‐γ for indicated time points, followed by medium collection for measuring nitrite production or cell lysis for SDS‐PAGE/immunoblotting or total RNA extraction for quantitative RT‐PCR.

### 2.7. Generation of Cav‐2 Knockout (KO) Mice

Cav‐2 KO mice in C57BL/6N background originating from Charles River Laboratories were generated with the assistance of Mouse Biology Program (UC Davis, CA). Briefly, the mouse Cav2 gene **(**MGI:107571) C57BL/6 deletion targeting vector was obtained from the KOMP vector repository, CHORI. The mouse Cav2 gene has three WT transcripts, of which all three are protein‐coding. Following removal of the floxed region of exons 2 and 3 of Cav2 gene, three transcripts are predicted to produce a truncated protein product. The Cav2 targeting vector (Design ID: DPGS00142_B_G09) was received as a glycerol stock, and DNA was quality control verified using five separate restriction digests prior to being linearized and prepared for electroporation using standard methods. Approximately 2.5 μg of the linearized targeting vector was electroporated into JM8A3.N1 ES cells derived from C57BL/6N mice according to a previously described strategy [30] and subjected to positive selection with G418. 48 clones selected with G418 were used to extract genomic DNA via proteinase K digestion at 75°C for 30 min in lysis buffer containing 10 mM tris/HCL pH8, 1 mM EDTA, 50 mM KCl, 2 mM MgCl_2_, and 3 mg/mL proteinase K. DNA was then screened using quantitative Taqman loss of allele via relative Ct method multiplexed in quadruplicate with the following oligos: Cav2 gene primers/probe include a forward primer CCGTGCAGACAATATGGAAGAGT, reverse primer CTGCGGCCCACACTTGTAC, probe 6‐Fam— ACAGACGTTGTCATTGGCCCATTGT—NFQ and were quantified next to a Y chromosome endogenous reference primers/probe including a forward primer GCCAGTCTGTGTCCCATCTC, reverse primer TGCCTGTATGTGATGGCATGT, probe VIC—TACAACCTTCTGCAGTGGGACAGGAACC—TAMRA. Nine clones with confirmed targeting were then expanded to a 6‐well plate and DNA was extracted via Qiagen blood and tissue kit according to manufacturer’s protocol. ~50 ng of DNA was tested by long range PCR of the 5′ arm using Invitrogen SequalPrep long PCR Kit according to manufacturer’s protocol with the following thermal cycling conditions: 94°C for 2 min; 10 cycles of 94°C for 15 s, 65°C for 15 s (↓1C/cycle), 68°C for 10 min; 25 cycles of 94°C for 15 s, 55°C for 30 s, 6–8°C for 10 min (+20 s/cycle), hold at 4°C. 5′ long range PCR included a genomic forward primer AGCAGGAAGATCCATCTAACCTGTGG paired with a cassette reverse primer GGTGGTGTGGGAAAGGGTTCGAAG for an expected 6.275 kbp targeted band. Long range PCR fragments were separated on a 0.8% agarose gel matrix at 120 v for 90 min and analyzed using a Kodak gel logic 200 instrument for expected 6.275 kbp targeted band detected in all nine clones. In addition, selection cassette copy number was confirmed as one copy/cell in a similar fashion as the Taqman LOA screening above using the same reference primers/probe with the following LacZ target primers/probe: forward primer ATCAGGATATGTGGCGGATGA, reverse primer TGATTTGTGTAGTCGGTTTATGCA, probe 6Fam—CGGCATTTTCCGTGACGTCTCGTT—TAMRA with eight clones confirmed to be LacZ positive. At least 20 spreads for three individual ES cell clones were chromosome counted to assess percent euploidy. All three euploid clones (50% or higher) were injected into C57BL/6N blastocysts, and 30 embryos/ES cell clone were implanted into two individual CD1 recipients. Resulting male chimeras were ranked by agouti coat color and mated with C57BL/6N WT females to identify the germline transmission of the F1 generation. DNA was extracted from ~3 mm tail snips using the Qiagen DNEasy blood and tissue kit according to the manufacturer’s instructions. DNA was then screened by PCR with the following primers: reverse primer *GTCTTATGGTCACAGCCAGATGAAGC*, cassette forward CACACCTCCCCCTGAACCTGAAAC. The Invitrogen SequalPrep long PCR kit was used according to manufacturer’s instructions. 20 μL PCR reactions included 0.4 μM of each primer with ~50 ng of template DNA. Thermal cycling included an initial denaturing at 94°C for 2 min; 10 cycles of 94°C for 15 s_,_ 65–55°C for 30 s (↓1°C/cycle), 68°C for 10 min; 25 cycles of 94°C for 15 s_,_ 55°C for 30 s, and 68°C for 10 min (+20 s/cycle); and maintained at 4°C. PCR reactions included a nontemplate control (ntc), negative WT control, and ~50ng of genomic DNA from ES cell clone as a positive control. PCR reactions were separated on a 1.5% agarose gel matrix at 120 v for 90 min and analyzed on a Kodak gel logic 200. Heterozygous female and male mice were selected for further breeding. The genotype of generated offspring was screened by PCR with the following thermal cycling conditions: denaturing at 94°C for 2 min; 10 cycles of 94°C for 15 s_,_ 65–55°C for 30 s (↓1°C/cycle), 68°C for 10 min; 25 cycles of 94°C for 15 s_,_ 55°C for 30 s, and 68°C for 10 min (+20 sec/cycle); and maintained at 4°C. To confirm the genotype, the following primers (with sequences shown in 5′‐3′ order) were used: (1) Cav‐2 sequence deletion forward primer (Cav2 KO‐F) CACACCTCCCCCTGAACCTGAAAC; (2) Cav‐2 common reverse primer (Cav2‐Com_R) GTCTGAGGAAGTTGCCCTTTGACC; (3) Cav‐2 WT forward primer (Cav2‐WT_F) GACAATATGGAAGAGTGTGACAGACG.

## 3. Results

### 3.1. Cav‐2 Is Regulated in a Time‐Dependent Manner in Primary Mouse Resident Peritoneal and RAW264.7 Macrophages Stimulated With LPS and IFN‐γ: Inverse Correlation of Cav‐2 With iNOS Expression Levels and NO Production

**Figure 1:**
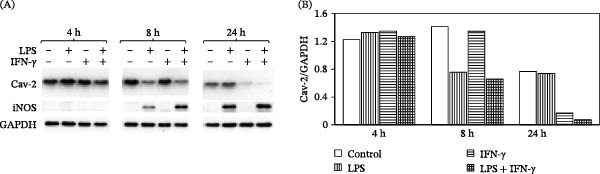
Time‐dependent effect of LPS and IFN‐γ on Cav‐2 and iNOS protein expression in primary mouse resident peritoneal macrophages. Primary resident peritoneal macrophages were isolated from C57BL6N mice as described in experimental procedures. Cells were treated without (−) or with (+) LPS (1 μg/mL), IFN‐γ (100 U/mL), or combination of LPS and IFN‐γ for 4, 8, and 24 h. Cells were lysed at indicated time points and processed for SDS‐PAGE and immunoblotting as described in experimental procedures. (A) Immunoblots with indicated antibodies. (B) Graphs representing densitometric ratios of Cav‐2 to GAPDH protein (loading control). Bars in (B) graph: blank = control; vertical = LPS; horizontal = IFN‐γ; grid = LPS + IFN‐γ.

Initially, we have examined if the expression of Cav‐2 protein is regulated by classical activators of macrophages known to induce iNOS and NO production. Specifically, mouse resident peritoneal macrophages were treated with LPS (1 μg/mL), IFN‐γ (100 U/mL), or a combination of LPS and IFN‐γ for 4, 8, and 24 h, followed by lysis and immunoblotting. Interestingly, LPS treatment transiently reduced by ca. 1.9‐fold Cav‐2 protein expression levels at the 8 h time point, while IFN‐γ had a delayed but more obvious ca. 4.6‐fold inhibitory effect at 24 h time point. Importantly, treatment with a combination of LPS and IFN‐γ resulted in the most robust downregulation of Cav‐2 protein expression by ca. 2.2‐ and 11‐fold at 8 and 24 h time points, respectively (Figure 1A; top immunoblot and Figure 1B). Remarkably, the degree of time‐dependent downregulation of Cav‐2 protein by a combination of LPS and IFN‐γ coincided with the increase in the expression levels of iNOS (Figure 1, 2^nd^ immunoblot from the top).

**Figure 2:**
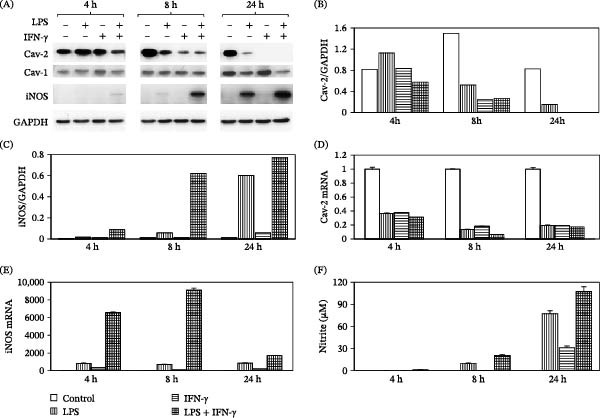
Time‐dependent effect of LPS and IFN‐γ on Cav‐2 and iNOS expression as well as nitrite production in RAW264.7 macrophages. RAW264.7 cells were plated at 3 x 10^5^ (A–C and F) per well of 12‐well plates or at 7.5 x 10^5^ per well of 6‐well plate (D, E) for 24 h, followed by medium change and treatment without (−) or with (+) LPS (100 ng/mL), IFN‐γ (100 U/mL), or combination of LPS and IFN‐γ for 4, 8, and 24 h. Medium was collected from 12‐well plates to determine nitrite accumulation and cells were lysed at indicated time points and processed for SDS‐PAGE and immunoblotting (A–C) or RNA isolation followed by qRT‐PCR (D, E) as described in experimental procedures. (A) Immunoblots with indicated antibodies. (B, C) Graphs representing densitometric ratios of Cav‐2 (B) and iNOS (C) to GAPDH protein (loading control). (D, E) Graphs representing quantitative real time PCR (qRT‐PCR) data for Cav‐2 mRNA (D) and iNOS mRNA (E) calculated based on the relative amount of target mRNA normalized to the endogenous reference GAPDH mRNA, expressed as fold change by LPS and IFN‐γ relative to control samples at each time point and represented as mean ± S.D. of three replications. (F) Graph representing time‐dependent nitrite accumulation in the media from RAW264.7 macrophages. The data are expressed as mean nitrite concentration ± S.D. (*n* = 3) from one representative out of a total of three experiments. Bars in (B–F) graphs: blank = control; vertical = LPS; horizontal = IFN‐γ; grid = LPS + IFN‐γ.

To determine if LPS and IFN‐γ‐induced regulation of Cav‐2 also occurs in a well‐characterized mouse macrophage cell line, RAW264.7, these cells were treated with LPS (100 ng/mL), IFN‐γ (100 U/mL), or a combination of LPS and IFN‐γ for 4, 8, and 24 h. Remarkably, all treatment conditions resulted in a gradual and time‐dependent reduction of Cav‐2 protein expression relative to nontreated control cells (Figure 2A; top immunoblot). However, reduction of Cav‐2 protein was most rapid in cells treated with a combination of LPS and IFN‐γ and was less robust in the presence of LPS alone compared to IFN‐γ alone or combination of the latter with LPS. Quantitative assessment of the densitometric ratios of Cav‐2/GAPDH (Figure 2B) established that while no inhibitory effect of LPS on Cav‐2 protein expression was observed at 4 h time point, ca. 2.9‐fold and 5.5‐fold reduction in Cav‐2 protein expression took place 8 and 24 h, respectively, after treatment with LPS (Figure 2A,B). Moreover, compared to LPS, IFN‐γ displayed a more robust inhibitory effect as it reduced the Cav‐2 protein expression by ca. 6.1‐fold at 8 h time point and no Cav‐2 protein could be detected at 24 h time point (Figure 2A,B). Finally, combined treatment with IFN‐γ and LPS resulted in the most rapid ca. 1.4‐fold reduction of Cav‐2 protein as early as at 4 h time point followed by ca. 5.5‐fold reduction and complete loss of Cav‐2 protein expression at 8 and 24 h time points, respectively (Figure 2A,B).

In a striking contrast to Cav‐2, Cav‐1 protein levels remained relatively constant at 4 and 8 h time points, while being noticeably reduced in cells treated with LPS and more so with a combination of LPS and IFN‐γ but not with IFN‐γ alone for 24 h (Figure 2A; 2^nd^ immunoblot from the top). Taken together, our data suggest that compared to Cav‐1, Cav‐2 is more rapidly and more robustly downregulated during RAW264.7 macrophage activation with LPS and IFN‐γ.

Notably, similar to primary mouse peritoneal macrophages, the degree of time‐dependent downregulation of Cav‐2 protein by a combination of LPS and IFN‐γ coincided with the increase in the expression levels of iNOS protein (Figure 2A, 3^rd^ immunoblot from the top and 2 C). Taken together, the inverse time‐dependent correlation between Cav‐2 and iNOS protein expression levels implies that the Cav‐2 protein could possibly regulate iNOS activation in macrophages stimulated with LPS and IFN‐γ.

To determine if previously observed time‐dependent LPS‐ and IFN‐γ‐induced decrease in Cav‐2 protein in RAW264.7 cells (Figure 2A; top immunoblots and Figure 2B) occurs at transcriptional level, we performed quantitative real time PCR with primers specific to mouse Cav‐2. Similar to protein, Cav‐2 mRNA levels were also reduced in LPS‐, IFN‐γ‐ and LPS/IFN‐γ‐treated cells in a time‐dependent manner (Figure 2D). However, in contrast to protein, LPS alone was just as rapid and robust as IFN‐γ in reducing Cav‐2 mRNA levels. Specifically, treatment with LPS (100 ng/mL) resulted in ca. 2.7‐, 7.3‐, and 5.1‐fold reduction in Cav‐2 mRNA at 4, 8, and 24 h time points, respectively (Figure 2D). Similarly to LPS, treatment with IFN‐γ (100 U/mL) decreased Cav‐2 mRNA levels by ca. 2.6‐, 5.5‐, and 5.3‐fold at 4, 8, and 24 h time points, respectively (Figure 2D). Combined treatment with LPS and IFN‐γ resulted in the greatest maximal reduction of Cav‐2 mRNA levels by ca. 3.2‐, 15.9‐, and 5.8‐fold at 4, 8, and 24 h time points, respectively (Figure 2D). Taken together, our RT‐PCR data based on the three time points examined, suggests that the maximal downregulation of Cav‐2 mRNA occurs 8 h after treatment with LPS and IFN‐γ. Similar to Cav‐2 and iNOS protein at 24 h time point (Figure 2A–C), the maximal downregulation of Cav‐2 mRNA by a combination of LPS and IFN‐γ coincided with the maximal upregulation of iNOS mRNA at 8 h time point (Compare Figure 2D vs. Figure 2E).

Overall, our quantitative RT‐PCR and immunoblotting data show that Cav‐2 is downregulated at mRNA and protein levels by LPS and IFN‐γ and that downregulation of Cav‐2 expression levels inversely correlates with the induction of iNOS expression by LPS and more so by a combination of LPS and IFN‐γ. Moreover, the inverse time‐dependent correlation between Cav‐2 and iNOS expression levels suggests that changes in Cav‐2 protein expression could possibly regulate iNOS expression and subsequent NO production in RAW264.7 cells stimulated with LPS and IFN‐γ.

### 3.2. NF‐κB p65 Is Rapidly Phosphorylated and Pharmacological Inhibition of NF‐κB Activation With PDCT Prevents Downregulation of Cav‐2 and Induction of iNOS in RAW264.7 Macrophages Stimulated With LPS and IFN‐γ

**Figure 3:**
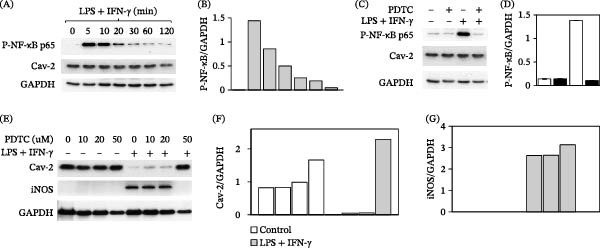
Effect of PDTC on Cav‐2, iNOS expression and phosphorylation levels of NF‐κB p65 at serine 536 in RAW264.7 macrophages. RAW264.7 cells were plated at 3 x 10^5^ per well of 12‐well plates for 24 h followed by indicated below various treatment conditions. To determine the time frame of detectable and maximal NF‐κB p65 phosphorylation at serine 536 (P‐NF‐kB), cells were treated with a combination of LPS (100 ng/mL) and IFN‐γ (100 U/mL) for indicated time points (0–120 min) (A, B). To confirm inhibitory effect of PDTC on NF‐κB activation, cells preincubated without (−) or with (+) PDTC (50 μM) for 1 h were further cotreated without (‐) or with (+) combination of LPS (100 ng/mL) and IFN‐γ (100 U/mL) for 5 min (C, D). To determine the effect of PDTC on Cav‐2 and iNOS expression, cells were preincubated without or with PDTC at indicated concentrations for 1 h followed by cotreatment without (−) or with (+) a combination of LPS (100 ng/mL) and IFN‐γ (100 U/mL) for 20 h (E‐G). (A, C, and E) Representative immunoblots with indicated antibodies. (B) Graph representing densitometric ratio of P‐NF‐κB to GAPDH determined based on immunoblots shown in (A). (D) Graph representing densitometric ratio of P‐NF‐kB to GAPDH determined based on immunoblots in (C). Bars: blank = control; filled = PDTC (50 μM). (F, G) Graphs representing densitometric ratios of Cav‐2 (F) and iNOS (G) to GAPDH protein (loading control) determined based on immunoblots shown in (E). The data with densitometric ratios (shown in B, D, F, and G) are expressed as mean ± S.D. of three independent sample loadings from one representative out of three experiments.

NF‐κB is the major signaling pathway involved in iNOS induction in macrophages [25]. Thus, we hypothesized that activation of the NF‐κB pathway would be involved in the observed downregulation of Cav‐2 by a combination of LPS and IFN‐γ. To determine possible involvement of the NF‐κB pathway in downregulation of Cav‐2, we examined activation of the NF‐κB pathway by LPS and IFN‐γ treatment by SDS‐PAGE and immunoblotting with anti‐P‐ NF‐κB p65 antibody. Treatment with LPS plus IFN‐γ resulted in a rapid and transient increase in phosphorylation levels of the NF‐κB p65 subunit (Figure 3A; top immunoblot and Figure 3B) before significant downregulation of Cav‐2 protein could be observed (Figure 3A; central immunoblot). Next, we examined the effect of PDTC (50 μM), which is a known inhibitor of NF‐κB activation [31] on NF‐κB p65 phosphorylation. Specifically, cells were preincubated with PDTC for 1 h followed by the treatment with LPS and IFN‐γ for 5 min time point at which the maximal phosphorylation of NF‐κB was observed (Figures 3A,B). Pretreatment with PDTC completely blocked LPS plus IFN‐γ‐induced phosphorylation of NF‐κB p65 (Figure 3C; top immunoblot and Figure 3D; filled bars). To directly test our hypothesis that NF‐κB is involved in downregulation of Cav‐2 by LPS and IFN‐γ, cells were pretreated with PDTC (10–50 μM) for 1 h followed by addition 20 h treatment without or with a combination of LPS and IFN‐γ. Remarkably, treatment with PDTC at 50 μM completely prevented LPS plus IFN‐γ‐induced downregulation of Cav‐2 (Figure 3E; top immunoblot; last lane and Figure 3F), suggesting that activation of the NF‐κB pathway is essential for Cav‐2 downregulation in activated macrophages. Consistent with previously observed inverse correlation between Cav‐2 and iNOS levels in LPS plus IFN‐γ‐stimulated primary macrophages (Figure 1) and RAW264.7 cells (Figure 2), prevention of Cav‐2 downregulation by PDTC (50 μM) was associated with a complete inhibition of iNOS induction (Figure 3E; 2^nd^ immunoblot; last lane and Figure 3G), further suggesting that NF‐κB‐dependent downregulation of Cav‐2 could possibly be involved in iNOS activation in macrophages stimulated with LPS and IFN‐γ. Taken together, our data with PDTC‐mediated blockade of Cav‐2 downregulation by LPS and IFN‐γ, suggests that activation of the NF‐κB pathway is essential for Cav‐2 downregulation and iNOS induction in LPS‐ and IFN‐γ‐stimulated macrophages.

### 3.3. Knockdown of Cav‐2 With RNAi Approach Increases iNOS Activation and STAT1 Phosphorylation in RAW264.7 Macrophages Stimulated With LPS and IFN‐γ

**Figure 4:**
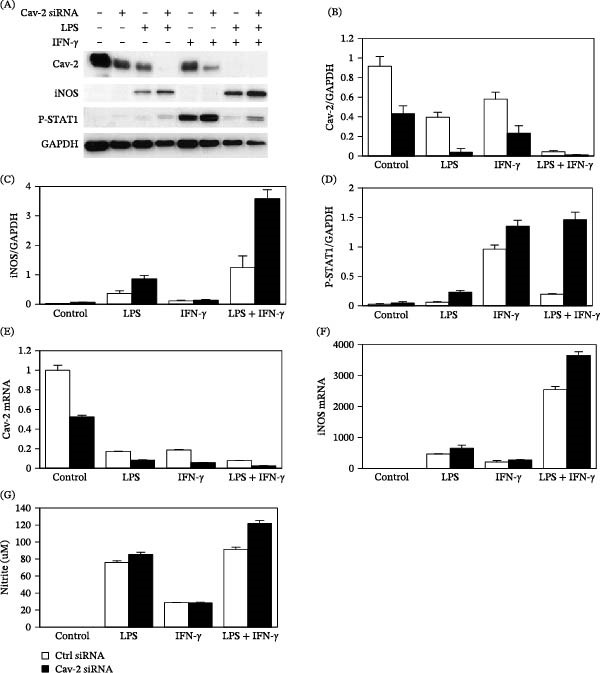
Effect of Cav‐2 siRNA on LPS‐ and IFN‐γ‐induced iNOS expression, nitrite production and STAT1 phosphorylation in RAW264.7 macrophages. RAW264.7 cells were transfected with nonsilencing control siRNA (Ctrl siRNA; blank bars) and with siRNA specifically targeting mouse Cav‐2 (Cav‐2 siRNA; filled bars) complexed with RNAiMAX transfection reagent (Invitrogen) according to manufacturer’s protocol. Next day, medium was changed and cells were incubated for additional 2 days. Three days after starting the transfection, cells were treated without (control) or with LPS (100 ng/mL), IFN‐γ (100 U/mL), or combination of LPS and IFN‐γ for 8 h (E, F; quantitative real time PCR) and 24 h (A; immunoblotting) and (G) (nitrite accumulation). (A) Representative immunoblots from control cells or cells stimulated with LPS and IFN‐γ for 24 h. (B–D) Graphs representing densitometric ratios of Cav‐2 (B), iNOS (C), and STAT1 phosphorylation at tyrosine 701 (P‐STAT1) (D) to GAPDH protein (loading control) based on immunoblots shown in (A) with the data expressed as mean ± S.D. of three independent sample loadings from one representative out of three experiments. (E, F) Representative quantitative PCR for Cav‐2 (E) and iNOS (F) with the data calculated based on the relative amount of target mRNA normalized to the endogenous reference GAPDH mRNA, expressed as fold change by 8 h stimulation with LPS and IFN‐γ relative to control samples and represented as mean ± S.D. of three replications. (G) Nitrite accumulation in the media from control cells and cells stimulated with LPS and IFN‐γ for 24 h. The data are expressed as mean nitrite concentration ± S.D. (*n* = 3) from one representative out of a total of three experiments.

To test directly our hypothesis that downregulation of Cav‐2 protein expression by LPS and IFN‐γ plays a role in regulating iNOS protein expression and iNOS‐dependent NO production in LPS‐ and IFN‐γ‐stimulated RAW264.7 macrophages, we compared the effect of siRNA specifically targeting murine Cav‐2 (Cav‐2 siRNA) as well as nonsilencing control siRNA on Cav‐2 and iNOS protein, mRNA and NO production determined by immunoblotting, quantitative RT‐PCR and spectrophotometric determination of nitrite accumulation, respectively. Cav‐2 siRNA reduced the Cav‐2 expression by ca. 2‐fold in control and more so in LPS‐, IFN‐γ‐, and LPS/IFN‐γ‐treated cells at protein (Figure 4A; top immunoblot and B) and mRNA levels (Figure 4E) compared to control siRNA. Importantly, siRNA knockdown of Cav‐2 resulted in ca. 2.3‐ and 2.9‐fold upregulation of iNOS protein expression in LPS‐ and LPS/IFN‐γ‐treated cells, respectively, but not in IFN‐γ‐treated cells (Figure 4A; 2^nd^ immunoblot from the top and 4 C). Consistent with its effect on iNOS protein levels, Cav‐2 siRNA also increased by ca. 1.38‐ and 1.43‐fold iNOS mRNA levels in LPS‐ and in LPS/IFN‐γ‐treated cells, respectively, but not in IFN‐γ‐treated cells compared to control siRNA (Figure 4F). Finally, consistent with the effect on iNOS protein and mRNA, siRNA knockdown of Cav‐2 increased by ca. 1.13‐ and 1.34‐fold nitrite accumulation in media from LPS‐ and from LPS/IFN‐γ‐treated cells, respectively, but not from IFN‐γ‐treated cells compared to Ctrl siRNA (Figure 4G).

Since STAT1 pathway is important in macrophage activation [32] including iNOS induction [25], we hypothesized that downregulation of Cav‐2 might also increase STAT1 activation in LPS and IFN‐γ‐treated cells. Interestingly, despite prolonged (24 h) treatment, there was still detectable STAT1 phosphorylation which was overall higher in cells treated with Cav‐2 siRNA relative to control siRNA (Figure 4A; 3^rd^ immunoblot from the top). Quantitative analysis revealed ca. 3.9‐, 1.4‐, and 7.4‐fold increase in STAT1 phosphorylation by Cav‐2 siRNA in cells stimulated with LPS, IFN‐γ, and LPS + IFN‐γ, respectively (Figure 4D). Taken together, our data using siRNA knockdown of Cav‐2 suggests that downregulation of Cav‐2 expression enhances STAT1 phosphorylation, iNOS expression, and subsequent NO production in RAW264.7 macrophages stimulated with LPS and a combination of LPS and IFN‐γ.

### 3.4. Primary Resident Peritoneal Macrophages Isolated From Cav‐2 KO Mice Display Enhanced iNOS Induction and NO Production in Response to IFN‐γ

**Figure 5:**
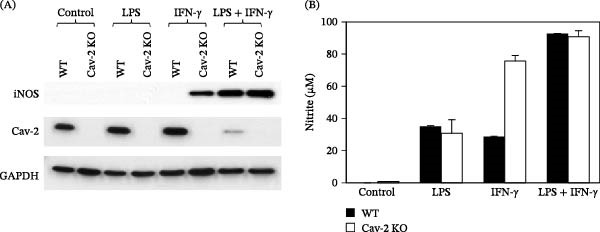
Effect of LPS and IFN‐γ on iNOS protein expression and nitrite production by primary resident peritoneal macrophages isolated from wild type and Cav‐2 knockout mice. Primary resident peritoneal macrophages were isolated from wild type and Cav‐2 KO C57BL6N mice as described in experimental procedures. Cells were treated without or with LPS (1 μg/mL), IFN‐γ (100 U/mL), or a combination of LPS and IFN‐γ for 24 h. Cells were lysed and processed for SDS‐PAGE and immunoblotting as described in experimental procedures. (A) Representative immunoblots with indicated antibodies. (B) Graph representing nitrite accumulation in the media from control and LPS‐ and IFN‐γ‐stimulated macrophages for 24 h. The data are expressed as mean nitrite concentration ± S.D. (*n* = 3) from one representative out of a total of two experiments.

To determine if Cav‐2 regulates iNOS and NO production in primary macrophages, freshly isolated resident peritoneal macrophages from WT and Cav‐2 KO mice were treated with LPS, IFN‐γ or a combination of LPS and IFN‐γ for 20 h. Remarkably, immunoblotting with iNOS antibody detected iNOS in IFN‐γ‐treated Cav‐2 KO but not WT macrophages (Figure 5A; top immunoblot; lane #6 vs. 5). No iNOS was detected in control and LPS treated cells (Figure 5; top immunoblot; Lanes #1–4) and comparable iNOS levels were detected in LPS plus IFN‐γ ‐stimulated WT and Cav‐2 KO macrophages (Figure 5; top immunoblot; Lanes #7 and 8). Consistent with iNOS protein, there was nearly a 3‐fold increase in nitrite accumulation in the media from Cav‐2 KO compared to WT macrophages stimulated with IFN‐γ but not with LPS or a combination of LPS and IFN‐γ (Figure 5B). Taken together, our data suggests that loss of Cav‐2 in primary mouse resident peritoneal macrophages enhances iNOS and subsequent NO production selectively in response to IFN‐γ but not to LPS nor a combination of LPS and IFN‐γ.

## 4. Discussion

The results of this study reveal that Cav‐2 expression is downregulated in primary mouse resident peritoneal macrophages and RAW264.7 macrophage cell line stimulated with known classical activators such as Gram‐negative bacterial LPS and a cytokine IFN‐γ. Using a pharmacological approach, we also show that LPS and IFN‐γ‐induced downregulation of Cav‐2 is NF‐κB‐dependent and that reduction or complete loss of Cav‐2 using siRNA or genetic deletion approaches may lead to enhanced iNOS and STAT1 activation.

While numerous studies have shown regulation of Cav‐1 expression in macrophages stimulated with LPS [33, 34] and IFN‐γ [34] and in other cell types [35], to the best of our knowledge, this is the first report of LPS‐ and IFN‐γ‐induced regulation of Cav‐2 expression in macrophages. Interestingly, thioglycollate‐elicited mouse peritoneal macrophages expressed higher levels of Cav‐2 protein and mRNA compared to their resident counterparts [26], suggesting the possibility of in vivo regulation of Cav‐2 in macrophages. Moreover, the most recent report has shown regulation of Cav‐2 in intestinal epithelial tissue during infection with *Salmonella typhimurium* [17], suggesting that Cav‐2 expression is regulated in epithelial and possibly other cell types vivo.

The fact that pharmacological inhibition of NF‐κB activation by PDTC prevents IFN‐γ and LPS‐induced downregulation of Cav‐2 suggests involvement of the NF‐κB pathway. This is the first experimental evidence supporting the involvement of NF‐κB in the regulation of Cav‐2 in any cell type. Interestingly, in contrast to NF‐κB‐dependent downregulation of Cav‐2 observed in our studies, Cav‐1 has been shown to be upregulated via NF‐κB‐dependent manner in LPS‐stimulated human lung microvascular endothelial cells [36]. Whereas these previously published studies also reported direct binding of NF‐κB to intronic NF‐κB consensus sites within the human Cav‐1 gene [36], to the best of our knowledge, there is no evidence for the presence of NF‐κB binding sites within the murine or human Cav‐2 gene. Thus, NF‐κB may downregulate Cav‐2 indirectly via a yet to be determined mechanism. One of the possible mechanisms could be NF‐κB‐dependent induction of microRNA (miRNA), which in turn could degrade Cav‐2 mRNA. Indeed, there is increasing evidence for NF‐κB‐dependent regulation of multiple miRNAs [37]. In addition, Cav‐2 itself has been shown to be targeted by at least three different miRNAs, including miR‐199a–3p [38], miR‐218 [8], and miR‐29a [17]. Nevertheless, none of these above‐mentioned miRNAs regulating Cav‐2 have been shown to be upregulated by NF‐κB pathway. Thus, additional studies examining the molecular mechanisms via which NF‐κB pathway downregulates Cav‐2 mRNA and protein are warranted.

Consistent with the inverse correlation between Cav‐2 and iNOS expression in LPS‐ and IFN‐γ‐stimulated RAW264.7 macrophage cell lines, the data showing that siRNA‐mediated knockdown of Cav‐2 is sufficient for a considerable increase in iNOS expression levels and nitrite production strongly supports involvement of Cav‐2 in negative regulation of iNOS expression and a consequent NO production. This negative role of Cav‐2 in regulating iNOS is further supported by a robust enhancement of iNOS activation in primary resident peritoneal macrophages isolated from Cav‐2 KO mice. However, dramatically enhanced iNOS activation in primary resident peritoneal macrophages isolated from Cav‐2 KO mice only occurs in response to IFN‐γ, which is in contrast to previously discussed RAW264.7 cell line treated with Cav‐2 siRNA. The latter difference may suggest that role of Cav‐2 in regulating primary macrophage versus RAW264.7 macrophage activation may depend on specific stimuli. Alternatively, the role of Cav‐2 may also depend on the specific source of macrophages. Indeed, there is a large body of experimental evidence showing that macrophages isolated from different tissues are very heterogeneous [39]. Thus, although beyond the scope of this study, it will be interesting to compare the role of Cav‐2 in primary macrophages isolated from various organs and tissues.

To the best of our knowledge, this is the first evidence for the role of Cav‐2 in regulating iNOS and STAT1 in macrophages or macrophage cell lines. In agreement with our data, recent studies determined increased iNOS expression levels and STAT1 phosphorylation in colon tissue and increased nitrite levels in peritoneal lavage from Cav‐2 KO mice treated with a lethal dose of LPS in vivo [18]. The authors of these previously mentioned studies concluded that this increased iNOS and STAT1 activation occurred in intestinal epithelial cells. However, no direct experimental evidence involving LPS‐stimulated purified colon epithelial cells, which could support the latter conclusion, was provided. Thus, in light of the previously published data in vivo with LPS injected mice [18] and our current data in vitro with RAW264.7 treated with Cav‐2 siRNA and primary peritoneal macrophages isolated from Cav‐2 KO mice, it is highly likely that loss of Cav‐2 will also enhance macrophage activation in vivo. Although beyond the scope of the current study, it will be very important to address the role of Cav‐2 in regulation of macrophage activation in vivo and how it translates to the outcome of various microbial and viral infections in which macrophage activation and innate immune response are of key significance.

The detailed signaling mechanisms responsible for regulation of iNOS by Cav‐2 in macrophages remain to be established. However, JAK/STAT1 pathway seems to be the most likely mechanism via which Cav‐2 regulates iNOS. This conclusion is supported by enhanced STAT1 phosphorylation in cells with downregulated Cav‐2. STAT1 was shown to be critical for transcriptional activation of iNOS in macrophages [25], including primary mouse peritoneal macrophages [40] and RAW264.7 stimulated with IFN‐γ and LPS [41]. It is also possible that in addition to the JAK/STAT1 mechanism via which Cav‐2 could regulate iNOS will play a role. For instance, NF‐κB or other pathways, which are involved in activation of iNOS in macrophages [25]. It will be important to conduct studies examining if Cav‐2 physically interact with STAT1 or its upstream kinase JAK and if Cav‐2 reexpression will directly lead to decreased STAT1 phosphorylation and thereby clarify the mechanistic link between NF‐κB‐mediated downregulation of Cav‐2 and subsequent STAT1‐mediated iNOS expression. Overall, future studies dissecting detailed signaling mechanisms involved in regulation of Cav‐2 and how exactly Cav‐2 regulates STAT1 and iNOS activation as well as pathophysiological significance are clearly warranted.

**Figure 6:**
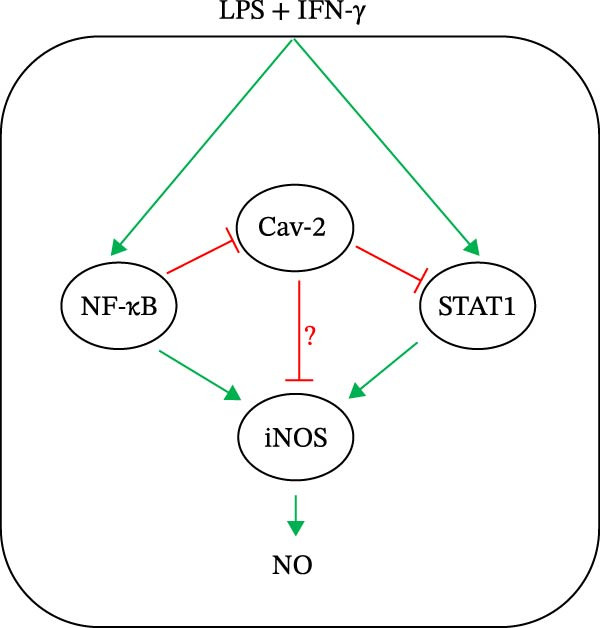
Proposed molecular mechanisms of Cav‐2 regulation and its involvement in mouse macrophage activation. Stimulation with LPS and IFN‐γ activates NF‐κB and STAT1 pathways. NF‐κB and STAT1 are important pathways in macrophage activation including iNOS activation and subsequent NO production. Activation of NF‐κB pathway leads to downregulation of Cav‐2 protein. Cav‐2 may suppress iNOS induction via its inhibitory effect on STAT1 activation and via additional yet to be identified pathways (?). Thus NF‐κB‐dependent downregulation of Cav‐2 may be an important molecular mechanism necessary for optimal macrophage activation. Therefore, Cav‐2 could serve as a molecular brake preventing excessive macrophage activation during inflammatory processes associated with various disorders including but not limited to microbial infections or sepsis.

In conclusion (summarized in Figure 6), we have discovered that Cav‐2 is downregulated during macrophage activation by LPS and IFN‐γ via NF‐κB‐dependent pathway(s). Moreover, we show that Cav‐2 downregulation may be required for full macrophage activation, evidenced by increased STAT1 and iNOS activation in cells with siRNA‐downregulated or genetically deleted Cav‐2. Thus, Cav‐2 may be an important regulator of macrophage activation via preventing excessive STAT1 and iNOS activation. Given importance of macrophage activation, in particular iNOS in various pathophysiological conditions [25, 42], our findings may have implications in better understanding and ultimately better management of various disorders, including infectious diseases, cancer, asthma, arthritis, multiple sclerosis, colitis, psoriasis, neurodegenerative diseases, transplant rejection, sepsis or septic shock.

## Funding

This research was supported by the National Heart, Lung, and Blood Institute (Grant R01‐HL081860 to Grzegorz Sowa) as well as the Department of Defense (Grants W81XWH‐15‐1‐0624 and W81XWH‐20‐1‐0688 to Grzegorz Sowa).

## Conflicts of Interest

The authors declare no conflicts of interest.

## Data Availability

The data are available upon request from the authors.
